# Discovering new bioactive molecules from microbial sources

**DOI:** 10.1111/1751-7915.12123

**Published:** 2014-03-24

**Authors:** Paolo Monciardini, Marianna Iorio, Sonia Maffioli, Margherita Sosio, Stefano Donadio

**Affiliations:** Naicons SrlMilano, Italy

## Abstract

There is an increased need for new drug leads to treat diseases in humans, animals and plants. A dramatic example is represented by the need for novel and more effective antibiotics to combat multidrug-resistant microbial pathogens. Natural products represent a major source of approved drugs and still play an important role in supplying chemical diversity, despite a decreased interest by large pharmaceutical companies. Novel approaches must be implemented to decrease the chances of rediscovering the tens of thousands of known natural products. In this review, we present an overview of natural product screening, focusing particularly on microbial products. Different approaches can be implemented to increase the probability of finding new bioactive molecules. We thus present the rationale and selected examples of the use of hypersensitive assays; of accessing unexplored microorganisms, including the metagenome; and of genome mining. We then focus our attention on the technology platform that we are currently using, consisting of approximately 70 000 microbial strains, mostly actinomycetes and filamentous fungi, and discuss about high-quality screening in the search for bioactive molecules. Finally, two case studies are discussed, including the spark that arose interest in the compound: in the case of orthoformimycin, the novel mechanism of action predicted a novel structural class; in the case of NAI-112, structural similarity pointed out to a possible *in vivo* activity. Both predictions were then experimentally confirmed.

## Introduction

In the search for new drug leads, particularly pressing is the need for novel and more effective antibiotics to combat the continuous surge in multidrug-resistant microbial pathogens. Aging, immunosuppression and invasive surgical procedures have increased the risk of contracting severe infections, while globalization is contributing to the rapid spread of multidrug-resistant pathogens. Consequently, increased morbidity, mortality and health-care costs are now due to infections caused by drug-resistant bacteria.

While genomic technologies and advances in automation have provided many novel targets and the ability to perform a large number of bioassays with limited human intervention in high-throughput screening (HTS), there is a general consensus that the chemical diversity offered by synthetic and combinatorial chemical libraries is not sufficient to provide the required number of quality hits for *hit-to-lead* programs in drug discovery. This has prompted many players in the field to advocate a return to screening natural products (Demain, 2014), which have provided and continue to provide a significant number of drugs approved for human use (Newman and Cragg, 2012). Significant advances in our understanding of biodiversity, in genomics and bioinformatics, and in synthetic biology have substantially changed our view of natural products and several tools are now available for effective discovery programs and for altering natural products by chemical and biological means (Fischbach and Walsh, 2009).

Within this context, it would be almost impossible to cover all topics relevant to natural product discovery with today's knowledge and tools. In order to limit the scope of this review, we will focus on bioactive molecules as parts of extracts, not as pure chemicals. We will also restrict ourselves to microbial products, our field of expertise. The reader is referred to previous descriptions of microbial bioprospecting (Bull, 2003) and on details on setting up screening programs for antibacterial and antifungal compounds (Donadio *et al*., 2009), topics that will not be covered here. Emphasis will be placed instead on the probabilistic nature of microbial product screening, using examples from the recent literature as well as our hands-on experience. Within this review, we will refer to bioactive compounds as ‘specialized metabolites’, a term believed more adequate than ‘secondary metabolites’ (Davies, 2013).

## Increasing the odds

During the golden era of microbial product screening, it can be estimated that, over a few decades, tens of millions of soil microorganisms were screened (Baltz, 2005), an enormous effort that provided the vast majority of microbial metabolites known today (Bérdy, 2005; 2012). While many details of those massive screening programs have not surfaced in the scientific literature (under the belief that the screening strategy was a sort of trade secret), we do know, however, that some metabolites were repeatedly rediscovered. This has led to estimates that the rate of discovery of different classes of microbial metabolites can vary significantly in ‘random’ screening campaigns (Baltz, 2007). This empirical knowledge has resulted in the general assumption that most of the low-hanging fruits have been already picked and few metabolites are left to be harvested unless massive screening programs are implemented (Zengler *et al*., 2002; Baltz, 2007).

**Figure 1:**
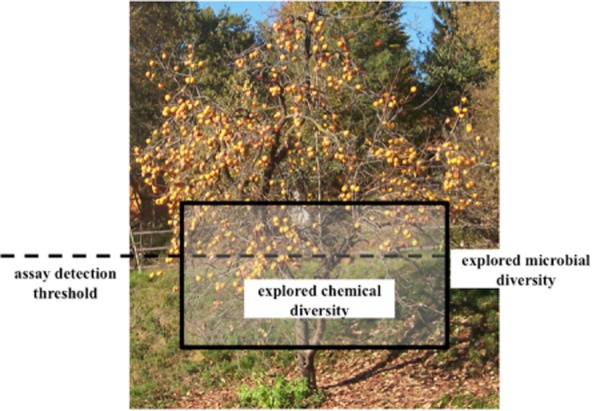
The concept of ‘low-hanging fruits’. Artistic rendering of the fraction of known metabolites arising from current explored microbial diversity and from the detection thresholds of the assays employed in screening. Tree photograph courtesy of Suzanne E. Ress.

Against this gloomy background, however, there are hopes for a ‘renaissance’ in natural product discovery (Baltz, 2008). Indeed, we are now aware that only a small fraction of the existing microbial diversity has been systematically sampled and that the production of specialized metabolites can occur in unexpected microbial taxa (Letzel *et al*., 2013). Similarly, the number of distinct biosynthetic pathways for specialized metabolites encoded within a single microbial genome is larger than the number of metabolites detected under ‘normal’ laboratory conditions. These observations suggest that we have so far sampled just a small fraction of the ‘parvome’, i.e. the set of small (Latin *parvus*) molecules produced by living organisms (Davies and Ryan, 2012). Thus, while ‘low-hanging fruits’ have been easier to reach starting from the ground, we simply need to change the sides from which we gather the fruits in order to unearth new bioactive compounds (Fig. 1).

**Figure 2:**
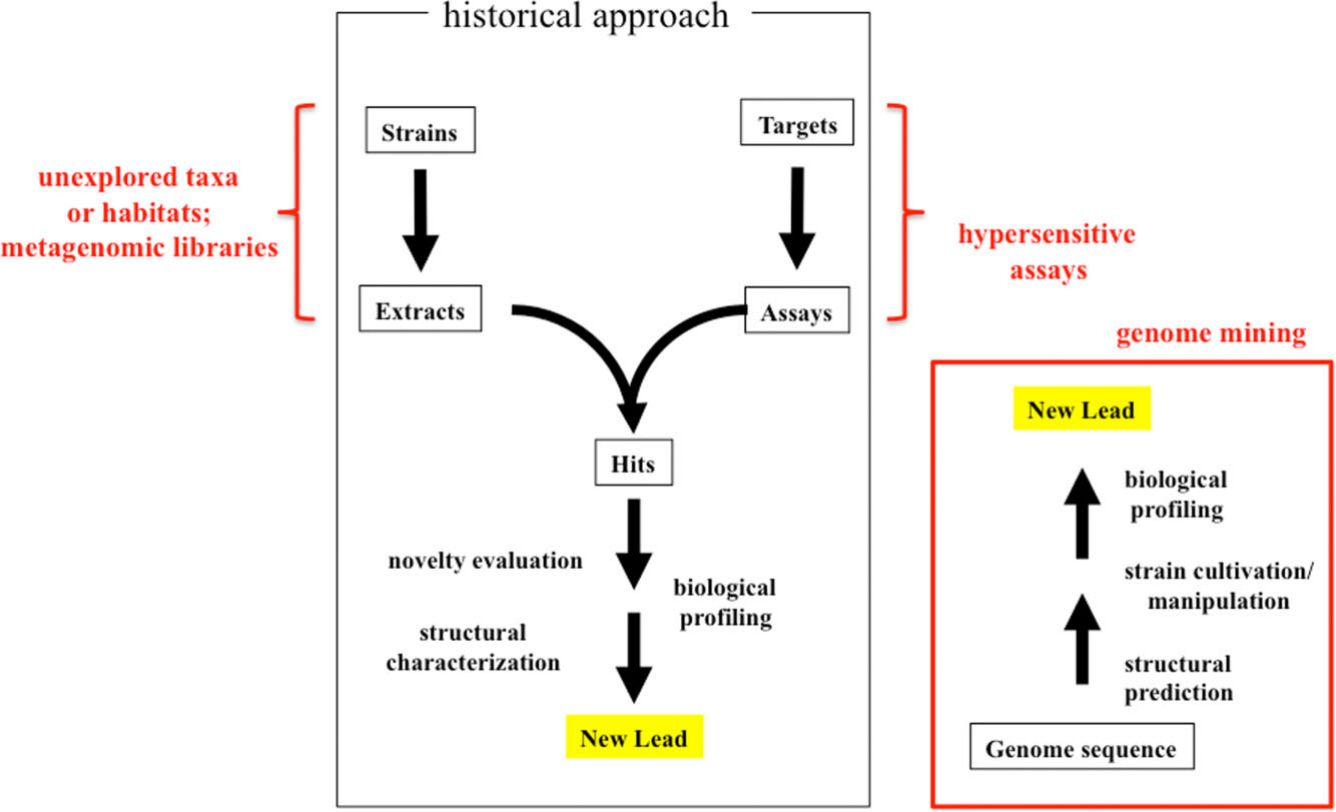
The historical approach to microbial product screening and possible improvements. The classical approach is depicted within the black rectangle. The red brackets denote approaches used to increase the probability of detecting new compounds within the ‘classical’ approach. The red rectangle denotes the paradigm shift used in genome mining. See text for details.

As mentioned, the large majority of known metabolites were discovered through empirical industrial screening programs whose details are unknown. However, a reasonable assumption is that easy-to-isolate strains were mostly evaluated for antimicrobial and/or cytotoxic activities, thus effectively selecting for those compounds that had either a high potency and/or were produced in significant amounts (Fig. 2). Therefore, two possible approaches can be undertaken to increase the probability of finding compounds not previously seen: (i) the use of detection methods with increased sensitivity and (ii) evaluating unexplored strains (Fig. 2). A drastic alternative consists of abandoning screening based on compound detection, and forcing instead strains to produce a desired specialized metabolite predicted from genomic sequence (Fig. 2).

In bioassay-guided screening programs, the introduction of an unexplored target or of a hypersensitive assay can increase the probability of detecting compounds not observed before (Fig. 2). In this case, however, it is important that the assay sees only a subset of possible bioactive compounds, i.e. it is target-specific; as an alternative, downstream tests can be implemented that can rapidly discard undesired compounds.

In principle, unexplored strains represent the best approach to discover novel chemistry (Fig. 2). This rests on the assumption that strain divergence (whether phylogenetic or ecological) has impacted on the divergence of biosynthetic pathways, resulting in some classes of specialized metabolites being more frequent or unique within certain taxa or habitats. However, the ability to produce specialized metabolites is more abundant in some microbial taxa than in others (Donadio *et al*., 2007), so not all unexplored strains may be equally productive. Furthermore, it is unrealistic to expect that unexplored strains will produce novel compounds only. In fact, some classes of known compounds are found also in such strains (e.g. Kim *et al*., 2006; Pozzi *et al*., 2011). This background noise must be empirically determined for each unexplored taxon/habitat.

A dramatic step in the use of unexplored strains is to bypass strain cultivation altogether: DNA directly from the environment can be introduced into a suitable expression host. Several of the formidable technical hurdles for this approach have been overcome (Ferrer *et al*., 2009). However, the approach works best when metagenomic libraries are made from environments enriched for strains that are good producers of specialized metabolites. Because of the many different metagenomes and of the role of the expression host, it might not be easy to establish the background of known compounds that can be encountered with metagenomic libraries. The reader is referred to recent, specialized reviews on the topic, which will not be further considered here (Banik and Brady, 2010; Piel, 2011).

In the last decade or so, a paradigm shift has been advocated for metabolite discovery: using the genome sequence as the starting point for going after specialized metabolites (Fig. 2). The diminishing cost of DNA sequencing and the development of bioinformatic tools for predicting the chemical classes of specialized metabolites from the sequence of the corresponding gene clusters have made it conceptually straightforward to predict the metabolite fingerprint encoded by each genome and compare it with that observed under wet-lab conditions (Corre and Challis, 2009). These studies have pointed out that a large portion of a strain's specialized metabolites is not routinely produced under laboratory conditions. However, getting these so-called cryptic molecules produced in sufficient amounts for structural and biological evaluation remains mostly a trial-and-error approach.

## High-throughput and high-quality screening

Independently of the approach undertaken, the objective of any search for bioactive molecules is the discovery of a new chemical entity or, alternatively, of a molecule possessing a previously unreported biological activity. Ideally, the new molecule should belong to a new chemical class, i.e. it has a mechanism of action different from that of structurally related compounds or it is not structurally related to molecules acting on the same target. Alternatively, the molecule must represent an improved variant within a known class, i.e. it possesses some advantages (e.g. potency, stability, amenability to semi-synthesis, etc.) with respect to structurally similar compounds. It should be noted that there is a higher probability of discovering an improved variant within chemical classes that have been poorly explored by screening, semi-synthesis and/or total synthesis. In contrast, within highly explored classes of microbial products (e.g. β-lactams, aminoglycosides, statins), such probability is small, unless a large number of variants can be rapidly evaluated.

The current screening paradigm is HTS, in which a large number of samples is evaluated for a desired signal, which should represent a rare event. It should be noted that HTS is best suited for exploring large libraries of substantially pure chemicals of known structure at a fixed concentration, thus allowing an immediate evaluation of the chemical nature of hits. In contrast, natural product screening usually involves complex extracts of unknown composition and concentration of active substance(s). Thus, HTS must be followed by extract fractionation and bioactivity identification against a background of known products. This limitation can be partially ameliorated by using pre-fractionated extracts, which however require a substantial investment (Tu and Yan, 2012). Furthermore, HTS relies on standardization of each step in the process, while natural product screening benefits from introducing diversity in the strains, growth conditions and method of extract preparation, which in turn result in increased noise in the screening.

We would like to introduce herein a different concept of screening, in which a sensibly smaller sample size is evaluated for a relatively frequent event. We designated this focused approach as High Quality Screening (HQS). There is obviously a whole grey area between HTS and HQS, but this arbitrary division may be useful to illustrate some concepts. In both cases, the objective of HTS and HQS is to identify a manageable number of hits for further characterization.

In HTS, great importance is placed on the assay, which has to be robust, amenable to automation and extremely specific, giving a low hit rate. Consequently, HTS requires a substantial investment in robotics and, for each screening program, the time required for assay automation and validation may exceed that for the screening campaign itself. At the same time, once this investment is made, HTS can process several projects with the same logics.

In HQS, a great emphasis is instead placed on the quality of the library, which must be highly enriched for the desired event. As a consequence, the library can be managed without extensive investment in automation or the need to develop sophisticated tests, which instead can be simple, whole-cell assays. However, in contrast to HTS, in which the same large library can be used for many different targets, each HQS program requires in principle a different library. A recently described application of HQS consists in the preselection of strains resistant to a particular bioactive molecule, followed by genetic probing for the expected biosynthetic cluster. This approach, applied to glycopeptides and ansamycins, has yielded interesting new variants of these well-known classes within a modest screening effort (Thaker *et al*., 2013). Since it is hypothesis-driven and does not require substantial investments, searching for new bioactive compounds by HQS may be attractive for academic laboratories and small companies.

In the following section, we will provide examples of new bioactive molecules discovered by following one of the general approaches illustrated below. We will then switch to our hands-on experience on discovering bioactive compounds.

## Use of hypersensitive assays

The widespread application of HTS has resulted in substantial improvements in assays, including sensitive methods for detection. With respect to screening microbial extracts, a significant step forward has been represented by whole-cell assays that can combine measuring the biological response of a cell to perturbation of selected target(s) within that cell. This approach has been particularly relevant in the search for antimicrobial compounds, in an effort to devise microbial cells hypersensitive to compounds acting on desired target(s). Among the many types of assays described in the literature, two general categories are present: reporter assays, which rely on the phenomenon that microbial cells can sense perturbation of a given cellular pathway at compound concentrations lower than those inhibiting growth (e.g. Fischer *et al*., 2004; Urban *et al*., 2007); and depletion assays, in which a microbial cell is made hypersensitive to inhibition of a desired target by artificially reducing its intracellular concentration (Roemer *et al*., 2003; Singh *et al*., 2007).

Among the hypersensitive assays, one that has been systematically applied to screening microbial extracts relies on the antisense technology developed for microbial pathogens. In this case, translation of the mRNA encoding a desired essential target is downregulated by expressing its cognate antisense RNA: A certain extent of target depletion does not have a significant impact on microbial growth but can make the cell hypersensitive to inhibitors of that target. One rapid way to screen microbial extracts consists in simultaneously testing, by agar diffusion, growth inhibition of the target strain, expressing the antisense RNA, versus growth inhibition of an isogenic control strain. A larger inhibition halo with the former strain suggests selective inhibition of the depleted target. This assay system has been systematically employed in HTS programs at Merck, resulting, for example, in the discovery of platensimycin, a selective inhibitor of fatty acid biosynthesis (Wang *et al*., 2006); of the thiazolyl peptide philipimycin and of lucensimycin (Zhang *et al*., 2008; Singh *et al*., 2009). It should be noted that some of these compounds are produced by relatively common strains, such as *Streptomyces platensis*, *Actinoplanes philippinensis* and *Streptomyces lucensis*, supporting the concept that hypersensitive assays can allow discovery of molecules that have escaped previous detection (Fig. 2).

## Accessing unexplored strains

Since only a small fraction of microorganisms has been systematically evaluated for the production of bioactive molecules, there is, in principle, a rich, untapped source for specialized metabolites. This has renewed interest in exploring additional microbial taxa, particular niches and/or poorly explored habitats such as marine sediments.

Among the underexplored bacterial taxa, myxobacteria certainly deserve the spotlight. Despite being long known as prolific producers of specialized metabolites, their systematic screening has been hampered by difficulties in their cultivation. Over the past decade or so, the systematic screening of myxobacteria has produced several new bioactive molecules (Wenzel and Müller, 2009). The thuggacins, thiazole-containing macrolides produced by *Sorangium cellulosum* and *Chondromyces crocatus*, exemplify a new chemical class of myxobacterial metabolites. These compounds show activity against *Mycobacterium tuberculosis*, possibly by interfering with the electron transport chain. Another example is represented by aurafuron, produced by *Stigmatella aurantiaca*.

Substantial efforts have also been devoted to exploring marine microbial strains as producers of bioactive molecules Fenical and Jensen ( 2006). While several of the marine strains are phylogenetically related to their terrestrial kin, the rationale is that marine habitats have selected for the production of compounds different from those selected by terrestrial habitats. Examples of novel or rare structural scaffolds discovered from marine strains include the abyssomicins, antibacterial compounds that interfere with folate metabolism produced by a *Verrucosispora* sp. (Bister *et al*., 2004); salinosporamide A, a potent proteasome inhibitor isolated from *Salinispora tropica* (Gulder and Moore, 2010); and the enediyne-derived cyanosporaside from *Salinispora pacifica* (Oh *et al*., 2006).

Symbioses, whether terrestrial or marine, are also promising ecological niches. Recent efforts to study microbial symbionts of insects, ascidians and fungi have yielded many new bioactive molecules. This topic has been recently reviewed in detail and will not be covered here (Piel, 2009; 2011).

## Genome mining

A decade ago, the first released genome sequences of *Streptomyces coelicolor* and *Streptomyces avermitilis* revealed that these soil-dwelling bacteria possess at least 20 biosynthetic gene clusters each, the majority of which could not be linked to compounds discovered in the previous 30–50 years of investigation. These initial observations were subsequently expanded, and it is now the norm to find that filamentous actinomycetes, myxobacteria and other prolific producers of specialized metabolites dedicate, on average, 5% of their large coding capacity to the synthesis of specialized metabolites. This has raised the intriguing possibility that one needs not search far: New molecules could first be bioinformatically identified from the genomic sequence of available strains and, knowing the type of compound to look for, a combination of targeted detection methods or simply screening different cultivation conditions would eventually lead to the identification of the predicted compounds. Following pioneering examples in the ‘pre-genomic era’ (Zazopoulos *et al*., 2003; McAlpine *et al*., 2005), this approach has now become straightforward, thanks also to the development of dedicated software (Blin *et al*., 2013).

Examples of new bioactive compounds accessed by genomic mining approaches include: stambomycin, a large macrolactone from *Streptomyces ambofaciens* with cytotoxic activity (Laureti *et al*., 2011); orfamide, a cyclic lipopeptide with antifungal activity produced by *Pseudomonas fluorescens* (Gross *et al*., 2007); the siderophore coelichelin from *Streptomyces coelicolor* (Lautru *et al*., 2005); and the morphogenic peptide catenulipeptin from *Catenulispora acidiphila* (Wang and van der Donk, 2012).

It should be noted that in some cases the molecule predicted from genome analysis was not produced under standard laboratory conditions. For example, stambomycin production could be observed only after overexpressing a pathway-specific regulator (Laureti *et al*., 2011; Aigle and Corre, 2012). These observations bode well for the future of this approach, since they suggest that many of these ‘silent’ clusters are biosynthetically functional and that the corresponding cryptic compounds have escaped detection during previous massive screening programs for the simple reason that they are not produced under standard laboratory conditions. The flip side of the coin is that triggering the expression of these gene clusters is a trial-and-error approach, with strategies such as manipulation of regulatory circuits in the native producer and/or heterologous expression attempted first (Gomez-Escribano and Bibb, 2014).

## Naicons technology

As mentioned, the search for new bioactive compounds has historically been biased towards the isolation and screening of the most common and easy-to-culture strains, although strain diversification was introduced with the aim of reducing the chances of rediscovering the same compounds. Up to the 1990s, however, only strains that produced a bioactive extract in ongoing programmes were kept and eventually characterized, resulting in lack of information on the ‘inactive’ strains.

With this historical background, the strain library owned by Naicons was generated in the period 1996–2005, with two major objectives: a focus on slow-growing and/or hard-to-isolate strains from two major groups of metabolite producers, the actinomycetes and the filamentous fungi; and the intent to use the strains for producing a 200 000 collection of microbial fermentation extracts to be used in HTS, mostly for antibacterial and antifungal projects. The strain library was thus put together by retrieving approximately 8000 strains from the historical Lepetit collection, which consisted mostly of *Actinoplanes* strains stored as producers of an antimicrobial activity (Lancini, 2006), and by *de novo* isolation of the remaining 60 000 strains, at an average pace of approximately 6000 strains per year. Different isolation methods were applied to a diverse collection of environmental samples (Lazzarini *et al*., 2000), and strain classification by morphological features was later flanked by the introduction of molecular methods (Donadio *et al*., 2002). In the last years, a 16S-guided isolation program was introduced, which allowed the systematic expansion of the library in previously uncultured or unclassified actinomycetes (Donadio *et al*., 2005), leading to the discovery of several new taxa (Busti *et al*., 2006a; Cavaletti *et al*., 2006; Monciardini *et al*., 2009) with the potential to produce specialized metabolites (Busti *et al*., 2006b).

**Figure 3:**
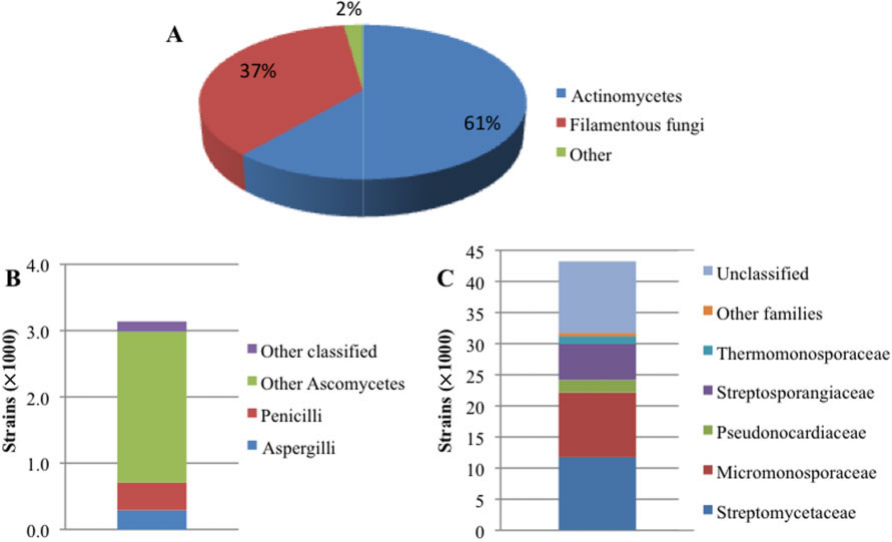
Composition of the Naicons strain library.
Percent distribution of the approximately 70 000 strains.High-level distribution of the approximately 3000 classified filamentous fungi.Suborder-level distribution of the approximately 43 000 actinomycetes.

As it stands today, the Naicons strain library contains just over 70 000 strains, mostly filamentous actinomycetes and filamentous fungi (Fig. 3). Among the filamentous fungi, most strains are represented by isolates that survived procedures for killing fast-germinating spores present in soil samples or that were retrieved from specialized habitats (plant endophytes, marine sediments, wild animal excrements). Only a fraction (12%) of the fungi are morphologically classified, and among them aspergilli and penicilli represent a minor part (Fig. 3B).

More information is available on the approximately 43 000 actinomycetes, 72% of which are classified according to their morphology (Fig. 3C). About 27% of the actinomycetes are classified as *Streptomyces*, a genus relatively easy to isolate from soil because of abundant sporulation and fast spore germination times. Among the other actinomycete families, the library is enriched in genera of the families *Micromonosporaceae* (∼10 000 isolates or 24% of the strains) and *Streptosporangiaceae* (∼5800 strains). Moreover, the collection contains > 1000 strains belonging to the recently described genus *Actinoallomurus* (Tamura *et al*., 2009), a genus shown to be a proficient producer of different classes of bioactive metabolites (Pozzi *et al*., 2011), including new molecules (Mazzetti *et al*., 2012).

It is not easy to compare Naicons strain library with public or private collections. Public collections, which were not assembled for screening purposes, typically consist of one or few strains for each species, in contrast to private collections that may contain several, independently isolated strains belonging to a single species. Few data are available on strain libraries at private institutions, with a notable example represented by Fundación Medina (http://www.medinadiscovery.com), whose strain library was also built with a focus on taxonomic diversification. The library includes approximately 58 000 actinomycetes, with about half of the strains belonging to several lineages other being *Streptomyces* (Genilloud *et al*., 2011). Overall, the two libraries at Medina and Naicons represent important, complementary tools to search for new bioactive molecules within the actinomycetes.

Most of the strains in the Naicons strain library were utilized to generate ∼160 000 microbial fermentation extracts by growing each strain under one to three different conditions and processing each culture by one or two extraction methods. The resulting extracts were used in several HTS projects, mostly for the identification of compounds with antibacterial or antifungal activity. All the HTS results were recorded in the dedicated, proprietary NPL database (Simone *et al*., 2013), which also includes information for each strain and details for extract preparation. Although not all HTS projects were run on the entire set of extracts, the NPL database includes data on 18 assays run on at least 10 000 extracts.

The database can thus be queried to retrieve information on the extracts that were active in a particular project and thus on the corresponding strains. This allows creating virtual sublibraries highly enriched in a particular bioactivity and/or arising from a desired group of strains. For example, actinomycetes produce selective antibacterial agents at a higher frequency than filamentous fungi, whose products tend to have general cytotoxic activities. Thus, an extract library derived from actinomycetes possessing antibacterial activity is expected to be enriched for selective antibacterial molecules. If such a library consisted of uncommon actinomycete genera, it would offer a reasonable probability of discovering new, selective antibacterial agents. When such an exercise was performed on the Naicons database, among the ∼76 000 actinomycete-derived extracts tested for growth inhibition of a *Staphylococcus aureus*, a subset of ∼2700 active strains was identified. Excluding about 25% strains for which no classification is available, the major taxa consist of 39% *Streptomyces*, 16% *Streptosporangineae* and 14% *Micromonosporineae*. Similar analyses were performed for other antimicrobial assays, leading to the identification of strains producing extracts with historical activity against fungi, Gram-negative and/or Gram-positive bacteria. These analyses are summarized in Table 1. In all cases, the active strains reflect the overall library composition, suggesting that the production of antimicrobial activities is a common trait among the actinomycete taxa included in the library.

**Table 1:** Actinomycete strains active in one or more HTS program^a^

Active strains	Gram+^b^	Gram-c	Fungi^D^
Total	6211	3542	1362
*Streptomyces*	2334	1553	627
Other classified	2454	1152	357
Not classified	1423	837	378

a The table reports the number of strains that produced an extract possessing growth inhibitory activity against the indicated group of pathogens.

b Activity against at least one of three *S. aureus*, two *Enterococcus faecium* or one *Streptococcus pyogenes* strains.

c Activity against at least one of four *E. coli*, one *Pseudomonas aeruginosa*, one *Acinetobacter baumannii* or one *Stenotrophomonas maltophilia* strains.

d Activity against at least one *Candida albicans* or one *Aspergillus fumigatus* strain; extracts likely to contain polyenes by a reversion assay have not been included.

The database can also be mined for desired target-based screening projects. A historical knowledge of the overall outcome of the screening program may help deciding whether a particular program is worth re-exploring. Indeed, in order to reduce the hit rate to a workable number of extracts for further analyses, pragmatical filters were implemented in larger HTS projects. With hindsight, some of these programs can be re-explored, focusing attention on a small number of extracts that can then be systematically evaluated for chemical novelty.

Below we report two examples of original chemical structures recovered from two data mining programs: orthoformimycin, which emerged from an HTS program aimed at bacterial translation inhibitors; and NAI-112, arising from a bacterial cell wall inhibitors program. In both cases, we present the discovery event in the context of the screening program, emphasizing the key observations that made the molecules worth pursuing.

## Case study 1: orthoformimycin

Significant effort was devoted to the development of an *in vitro* protein synthesis assay that would allow the detection of compounds affecting any step in translation (Brandi *et al*., 2007). The assay system made use of an identical, *in vitro* generated mRNA that could be translated with comparable efficiency by either a bacterial or a eukaryotic cell-free extract. This HTS program yielded GE81112, the first-in-class specific, selective tetrapeptide inhibitor of bacterial translation initiation (Brandi *et al*., 2006a), as well as a cyclic peptide (Brandi *et al*., 2006b) later established to be a variant of dityromycin (Brandi *et al*., 2012).

However, the assay had a relatively high hit rate and was actually enriching for novel compounds with high *in vitro* activity but with little or no activity against whole bacterial cells. It was then decided that only those extracts showing selective inhibition of bacterial protein synthesis and antibacterial activity would be evaluated for chemical novelty. One such extract derived from *Streptomyces* sp. ID107558 (Maffioli *et al*., 2013).

**Figure 4:**
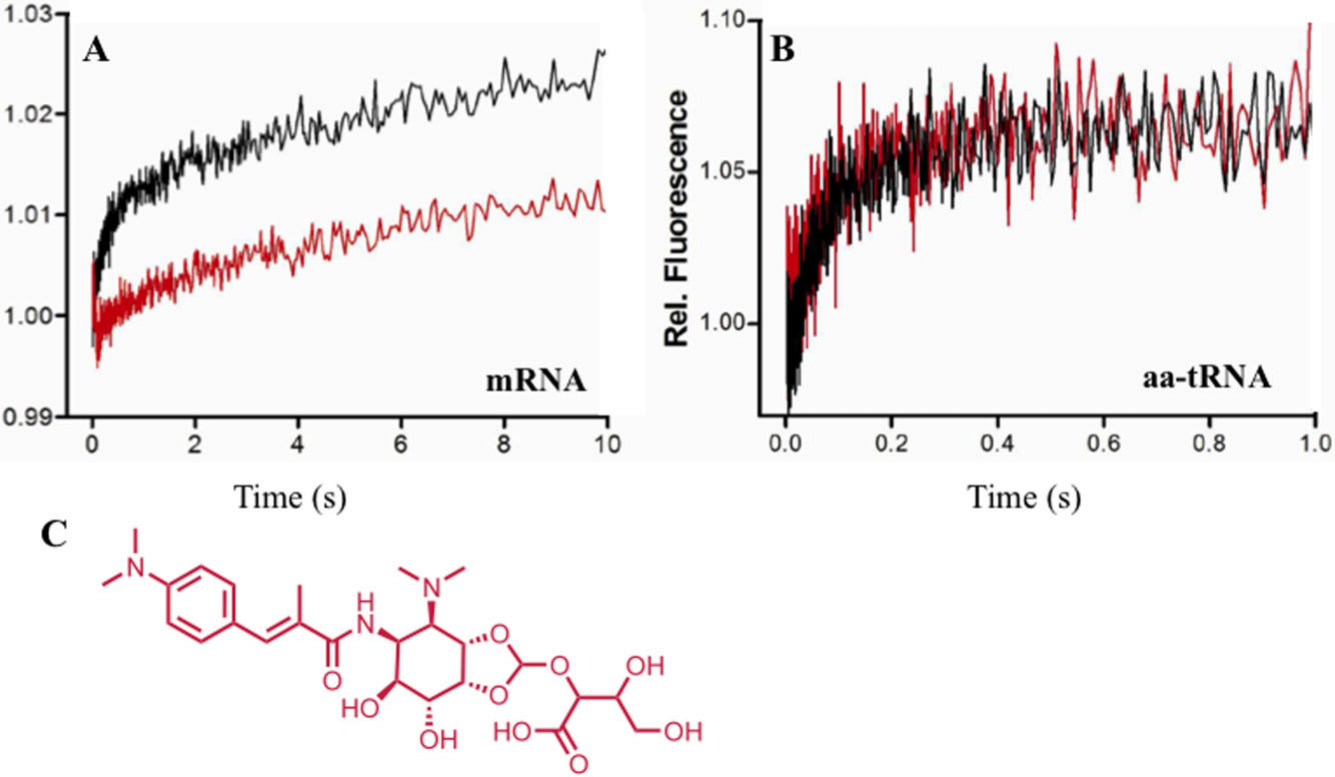
Discovery of orthoformimycin.
Fluorescence stopped-flow kinetics of the mRNA andPhe-tRNA EF-G-dependent translocation in the absence (black trace) or in the presence of 20 μM of orthoformimycin (red trace).Structure of orthoformimycin. Adapted from Maffioli and colleagues (2013).

Despite its weak antibacterial activity, this extract showed a substantial and selective inhibition of protein synthesis *in vivo* in both *Bacillus subtilis* and *Escherichia coli*. *In vitro*, the extract bound to the 50S ribosomal subunit in its free form and, with a somewhat lower affinity, with part of the 70S complex. Upon further investigation, it turned out that the purified active molecule interfered with translocation in a way different from that of other known translocation inhibitors like tetracycline, puromycin and GE82832. The compound did not interfere with the ribosomal binding of EF-G, but it strongly inhibited the EF-G-dependent movement of the mRNA on the ribosome (Fig. 4A), as well as Pi release from EF-G. Surprisingly, it did not affect EF-G dependent aminoacyl-tRNA translocation (Fig. 4B). The unique mechanism of action observed for the metabolite produced by strain ID107558 suggested a novel chemical structure, which prompted further studies on this molecule (Maffioli *et al*., 2013).

Structure elucidation of the active molecule (Fig. 4C) resulted in the first example of a natural product containing an orthoformate moiety. While orthoesters are encountered in specialized metabolites from fungi, plants and bacteria, an orthoformate is, to our knowledge, unique. Accordingly, the compound was named orthoformimycin (Maffioli *et al*., 2013).

In this example, the novelty of the mechanism of action predicted a structural novelty for the compound, a prediction that was experimentally confirmed, yielding some completely novel features. It should be noted that many different classes of microbial metabolites interfere with translation, and details of their mechanisms of action are known for many of them (Gualerzi *et al*., 2013). This knowledge facilitated predicting a new chemical class from a novel mechanism of action.

## Case study 2: NAI-112

The second example deals with a phenotypic assay for discovering bacterial cell wall inhibitors. The screening program, whose details have been described elsewhere (Jabés and Donadio, 2010), aimed at identifying all classes of bacterial cell wall inhibitors except for beta-lactams and glycopeptides, which were discarded by reversion assays. For reasons that are not yet understood, it turned out that the screening was in fact highly selective for lantibiotics, and led to the discovery of the potent lantibiotic NAI-107 (Castiglione *et al*., 2008), currently under late preclinical studies (Jabés *et al*., 2011), as well as the related but less active 97518 (Maffioli *et al*., 2009) and the improved actagardin variant NAI-802 (Simone *et al*., 2013).

Because the screening program was highly selective for lantibiotics, a class poorly explored within the actinomycetes, we reasoned that there was a relatively high probability of discovering novel compounds by evaluating those hits that, according to our database, had not been structurally characterized. One such extract was derived from *Actinoplanes* sp. ID112781 (Iorio *et al*., 2014).

**Figure 5:**
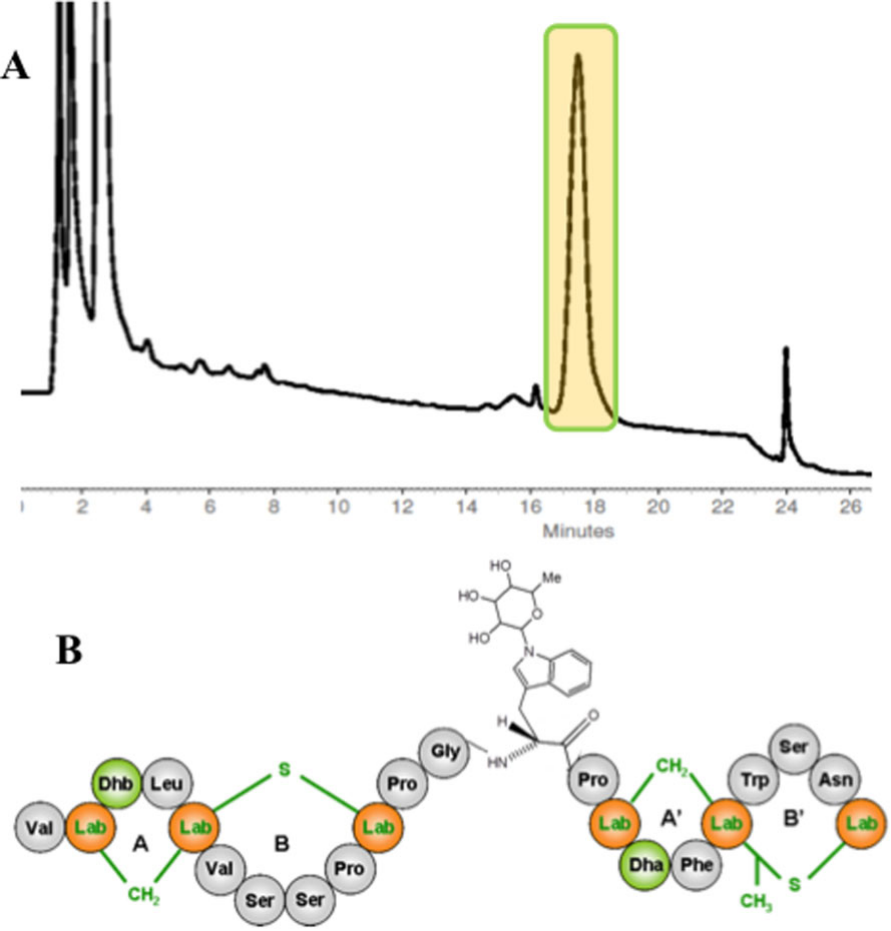
Discovery of NAI-112.
HPLC trace of the fractionated extract from *Actinoplanes* ID112781. The only peak with antibacterial activity is highlighted.Chemical structure of NAI-112. The labionine (Lab) rings AB and A′B′ are highlighted, together with dehydrated amino acids Dha (dehydroalanine) and Dhb (dehydrobutyrine). The *N*-glycosylated Trp residue is detailed. Adapted from Iorio *et al*. (2014).

The extract prepared from this strain exhibited weak antibacterial activity, which was associated to a single peak upon high-performance liquid chromatography (HPLC) fractionation (Fig. 5) and designated as NAI-112. The corresponding high resolution mass spectrometry (MS) was consistent with the formula C_109_H_147_N_25_O_31_S_2_ and a molecular weight of 2366 amu, suggesting a lanthipeptide (Arnison *et al*., 2013). Consistently, specific chemical reactions demonstrated the presence of one amino and one carboxylic group, as well as two thioethers and two dehydrated amino acids. Surprisingly, under mild acidic conditions, NAI-112 lost a hydrophilic moiety of 146 amu, which was successively identified as a 6-deoxyhexose (Fig. 5). However, the NMR spectrum of intact NAI-112 contained many overlapping signals, while no step-wise fragmentation was observed upon MS fragmentation, thus preventing a partial amino acid sequence of the peptide (Iorio *et al*., 2014). Luckily, weak acidic treatment led to the isolation of the [1–13] N-terminal, deglycosylated fragment of NAI-112, whose primary structure could be partially established by nuclear magnetic resonance (NMR) and used to query a draft genome of the *Actinoplanes* strain. This approach led to the identification of the entire precursor peptide of NAI-112, encoded within a biosynthetic gene cluster with features typical for a class III lanthipeptide, but which also encoded a glycosyltransferase, as expected. By combining NMR data, MS fragmentations and the gene sequence, the structure of NAI-112 was established as a 22-aa, neutrally charged lanthipeptide containing *N*-terminal labionin and *C*-terminal methyl-labionin residues separated by a 4-aa linker, decorated with an *N*-glycosylated Trp residue. It should be noted that NAI-112 presents two structural features unprecedented in lanthipeptides: a methyl-labionin, akin to the more frequent methyl-lanthionine (Arnison *et al*., 2013), and a glycosylation. Furthermore, there are just few examples of natural products carrying a glycosyl moiety on the indole nitrogen of tryptophan.

Despite that NAI-112 derived from a screening for bacterial cell wall inhibitors, its weak antibacterial activity and unusual structural features prompted us to look for other activities. Inspired by the recent report that labyrinthopeptin, another labionin-containing class III lanthipeptide, was effective in animal models of neuropathic pain (Meindl *et al*., 2010), NAI-112 was thus tested for its potential pain-alleviating effects: It showed marked activity in both the formalin and the chronic nerve injury models, suggesting a broad activity against acute and inflammation pain (Iorio *et al*., 2014).

In this case study, it was the structural class that suggested a possible bioactivity for the compound, which was then experimentally confirmed. In contrast with bacterial translation inhibitors, there was only one precedent for a class III lanthipeptide active against neuropathic pain. It remains to be determined whether such activity represents a general feature of labionin-containing lanthipeptides or if NAI-112 activity must be considered a lucky strike.

## Outlook

The examples reported above represent a small and somehow arbitrary selection of bioactive molecules discovered over the past decade. They clearly indicate that there are plenty of opportunities to discover new compounds from microbial sources, including new chemical classes or more potent variants of known classes. In most, if not all cases, new molecules were discovered by applying ‘smart screens’ of some sort.

It should also be noted that natural product screening has dramatically changed: It is now mostly a business of academia and small biotech companies, with few notable exceptions of large companies still active in the field; in contrast, a couple of decades ago it was the drug discovery arena of most large companies, with only a few academic groups interested in the field. The resurgent interest from academia has certainly contributed to the development of innovative tools, including the ability to detect bioactive molecules directly on microbial colony surfaces (Esquenazi *et al*., 2009). We are thus confident that the discovery of new specialized metabolites, as well as understanding their role in nature, will continue to be a scientifically rewarding activity.

One tool that has enormously contributed to rejuvenate microbial product screening is undoubtedly genomics. Whether used directly in genome mining approaches or to assist in structure elucidation, the previously separate disciplines of natural product chemistry and microbial physiology are becoming increasingly intertwined because of genomics. Furthermore, the ability to provide immediate access to the enzymes involved in the biosynthesis of specialized metabolites (Medema *et al*., 2011) offers further opportunities for generating improved derivatives of bioactive molecules. We are thus confident that the near future will continue to provide an abundant harvest of new bioactive molecules from microbial sources.

## References

[b1] Aigle B, Corre C (2012). Waking up *Streptomyces* secondary metabolism by constitutive expression of activators or genetic disruption of repressors. Methods Enzymol.

[b2] Arnison PG, Bibb MJ, Bierbaum G, Bowers AA, Bugni TS, Bulaj G (2013). Ribosomally synthesized and post-translationally modified peptide natural products: overview and recommendations for a universal nomenclature. Nat Prod Rep.

[b3] Baltz RH (2005). Antibiotic discovery from actinomycetes: will a renaissance follow the decline and fall?. SIM News.

[b4] Baltz RH (2007). Antimicrobials from actinomycetes: back to the future. Microbe.

[b5] Baltz RH (2008). Renaissance in antibacterial discovery from actinomycetes. Curr Opin Pharmacol.

[b6] Banik JJ, Brady SF (2010). Recent application of metagenomic approaches toward the discovery of antimicrobials and other bioactive small molecules. Curr Opin Microbiol.

[b7] Bérdy J (2005). Bioactive microbial metabolites. J Antibiot.

[b8] Bérdy J (2012). Thoughts and facts about antibiotics: where we are now and where we are heading. J Antibiot.

[b9] Bister B, Bischoff D, Ströbele M, Riedlinger J, Reicke A, Wolter F (2004). Abyssomicin C-A polycyclic antibiotic from a marine *Verrucosispora* strain as an inhibitor of the p-aminobenzoic acid/tetrahydrofolate biosynthesis pathway. Angew Chem Int Ed Engl.

[b10] Blin K, Medema MH, Kazempour D, Fischbach MA, Breitling R, Takano E, Weber T (2013). antiSMASH 2.0 – a versatile platform for genome mining of secondary metabolite producers. Nucleic Acids Res.

[b13] Brandi L, Fabbretti A, La Teana A, Abbondi M, Losi D, Donadio S, Gualerzi CO (2006a). Specific, efficient, and selective inhibition of prokaryotic translation initiation by a novel peptide antibiotic. Proc Natl Acad Sci USA.

[b11] Brandi L, Fabbretti A, Di Stefano M, Lazzarini A, Abbondi M, Gualerzi CO (2006b). Characterization of GE82832, a peptide inhibitor of translocation interacting with bacterial 30S ribosomal subunits. RNA.

[b14] Brandi L, Fabbretti A, Milon P, Carotti M, Pon CL, Gualerzi CO (2007). Methods for identifying compounds that specifically target translation. Methods Enzymol.

[b15] Brandi L, Maffioli S, Donadio S, Quaglia F, Sette M, Milón P (2012). Structural and functional characterization of the bacterial translocation inhibitor GE82832. FEBS Lett.

[b16] Bull AT (2003). Microbial Diversity and Bioprospecting.

[b17] Busti E, Cavaletti L, Monciardini P, Schumann P, Rohde M, Sosio M, Donadio S (2006a). *Catenulispora acidiphila* gen. nov., sp. nov., a novel mycelium-forming actinomycete and proposal of *Catenulisporaceae* fam. nov. Int J Syst Evol Microbiol.

[b18] Busti E, Monciardini P, Cavaletti L, Bamonte R, Lazzarini A, Sosio M, Donadio S (2006b). Antibiotic-producing ability by representatives of a newly discovered lineage of actinomycetes. Microbiology.

[b19] Castiglione F, Lazzarini A, Carrano L, Corti E, Ciciliato I, Gastaldo L (2008). Determining the structure and mode of action of microbisporicin, a potent lantibiotic active against multiresistant pathogens. Chem Biol.

[b20] Cavaletti L, Monciardini P, Schumann P, Rohde M, Bamonte R, Busti E (2006). *Actinospica acidiphila* gen. nov., sp. nov., and *Actinospica robiniae* gen. nov., sp. nov.; proposal for *Actinospicaceae* fam. nov. and *Catenulisporinae* subordo nov. in the order *Actinomycetales*. Int J Syst Evol Microbiol.

[b21] Corre C, Challis GL (2009). New natural product biosynthetic chemistry discovered by genome mining. Nat Prod Rep.

[b22] Davies J (2013). Specialized microbial metabolites: functions and origins. J Antibiot.

[b23] Davies J, Ryan KS (2012). Introducing the parvome: bioactive compounds in the microbial world. ACS Chem Biol.

[b24] Demain AL (2014). Importance of microbial natural products and the need to revitalize their discovery. J Ind Microbiol Biotechnol.

[b25] Donadio S, Monciardini P, Alduina R, Mazza P, Chiocchini C, Cavaletti L (2002). Microbial technologies for the discovery of novel bioactive metabolites. J Biotechnol.

[b26] Donadio S, Busti E, Monciardini P, Bamonte R, Mazza P, Sosio M, Cavaletti L (2005). Sources of polyketides and non-ribosomal peptides. Ernst Schering Res Found Workshop.

[b27] Donadio S, Monciardini P, Sosio M (2007). Polyketide synthases and nonribosomal peptide synthetases: the emerging view from bacterial genomics. Nat Prod Rep.

[b28] Donadio S, Monciardini P, Sosio M (2009). Approaches to discovering novel antibacterial and antifungal agents. Methods Enzymol.

[b29] Esquenazi E, Yang YL, Watrous J, Gerwick WH, Dorrestein PC (2009). Imaging mass spectrometry of natural products. Nat Prod Rep.

[b30] Fenical W, Jensen PR (2006). Developing a new resource for drug discovery: marine actinomycete bacteria. Nat Chem Biol.

[b31] Ferrer M, Beloqui A, Timmis KN, Golyshin PN (2009). Metagenomics for mining new genetic resources of microbial communities. J Mol Microbiol Biotechnol.

[b32] Fischbach MA, Walsh CT (2009). Antibiotics for emerging pathogens. Science.

[b33] Fischer HP, Brunner NA, Wieland B, Paquette J, Macko L, Ziegelbauer K, Freiberg C (2004). Identification of antibiotic stress-inducible promoters: a systematic approach to novel pathway-specific reporter assays for antibacterial drug discovery. Genome Res.

[b34] Genilloud O, Gonzalez I, Salazar O, Martin J, Tormo JR, Vicente F (2011). Current approaches to exploit actinomycetes as a source of novel natural products. J Ind Microbiol Biotechnol.

[b35] Gomez-Escribano JP, Bibb MJ (2014). Heterologous expression of natural product biosynthetic gene clusters in *Streptomyces coelicolor*: from genome mining to manipulation of biosynthetic pathways. J Ind Microbiol Biotechnol.

[b36] Gross H, Stockwell VO, Henkels MD, Nowak-Thompson B, Loper JE, Gerwick WH (2007). The genomisotopic approach: a systematic method to isolate products of orphan biosynthetic gene clusters. Chem Biol.

[b37] Gualerzi CO, Brandi L, Fabbretti A, Pon CL (2013). Antibiotics: Targets, Mechanisms and Resistance.

[b38] Gulder TA, Moore BS (2010). Salinosporamide natural products: potent 20S proteasome inhibitors as promising cancer chemotherapeutics. Angew Chem Int Ed Engl.

[b39] Iorio M, Sasso O, Maffioli SI, Bertorelli R, Monciardini P, Sosio M (2014). A glycosylated, labionin-containing lanthipeptide with marked antinociceptive activity. ACS Chem Biol.

[b40] Jabés D, Donadio S (2010). Strategies for the isolation and characterization of antibacterial lantibiotics. Methods Mol Biol.

[b41] Jabés D, Brunati C, Candiani G, Riva S, Romanó G, Donadio S (2011). Efficacy of the new lantibiotic NAI-107 in experimental infections induced by multidrug-resistant Gram-positive pathogens. Antimicrob Agents Chemother.

[b42] Kim TK, Hewavitharana AK, Shaw PN, Fuerst JA (2006). Discovery of a new source of rifamycin antibiotics in marine sponge actinobacteria by phylogenetic prediction. Appl Environ Microbiol.

[b43] Lancini G (2006). Forty years of antibiotic discovery at Lepetit: a personal journey. SIM News.

[b44] Laureti L, Song L, Huangm S, Correm C, Leblond P, Challis GL, Aigle B (2011). Identification of a bioactive 51-membered macrolide complex by activation of a silent polyketide synthase in *Streptomyces ambofacien*s. Proc Natl Acad Sci USA.

[b45] Lautru S, Deeth RJ, Bailey LM, Challis GL (2005). Discovery of a new peptide natural product by *Streptomyces coelicolor* genome mining. Nat Chem Biol.

[b46] Lazzarini A, Cavaletti L, Toppo G, Marinelli F (2000). Rare genera of actinomycetes as potential producers of new antibiotics. Antonie Van Leeuwenhoek.

[b47] Letzel AC, Pidot SJ, Hertweck C (2013). A genomic approach to the cryptic secondary metabolome of the anaerobic world. Nat Prod Rep.

[b48] McAlpine JB, Bachmann BO, Piraee M, Tremblay S, Alarco AM, Zazopoulos E, Farnet CM (2005). Microbial genomics as a guide to drug discovery and structural elucidation: ECO-02301, a novel antifungal agent, as an example. J Nat Prod.

[b49] Maffioli S, Potenza D, Vasila F, De Matteo M, Sosio M, Marsiglia B (2009). Structure revision of the lantibiotic 97518. J Nat Prod.

[b50] Maffioli SI, Fabbretti A, Brandi L, Savelsbergh A, Monciardini P, Abbondi M (2013). Orthoformimycin, a selective inhibitor of bacterial translation elongation from *Streptomyces* containing an unusual orthoformate. ACS Chem Biol.

[b51] Mazzetti C, Ornaghi M, Gaspari E, Parapini S, Maffioli S, Sosio M, Donadio S (2012). Halogenated spirotetronates from *Actinoallomurus*. J Nat Prod.

[b52] Medema MH, Breitling R, Bovenberg R, Takano E (2011). Exploiting plug-and-play synthetic biology for drug discovery and production in microorganisms. Nat Rev Microbiol.

[b53] Meindl K, Schmiederer T, Schneider K, Reicke A, Butz D, Keller S (2010). Labyrinthopeptins: a new class of carbacyclic lantibiotics. Angew Chem Int Ed.

[b54] Monciardini P, Cavaletti L, Ranghetti A, Schumann P, Rohde M, Bamonte R (2009). Two novel members of the family *Micromonosporaceae, Rugosimonospora acidiphila* gen. nov, sp. nov. and *Rugosimonospora africa*na sp. nov. Int J Syst Evol Microbiol.

[b55] Newman DJ, Cragg GM (2012). Natural products as sources of new drugs over the 30 years from 1981 to 2010. J Nat Prod.

[b56] Oh DC, Williams PG, Kauffman CA, Jensen PR, Fenical W (2006). Cyanosporasides A and B, chloro-and cyano-cyclopenta[a]indene glycosides from the marine actinomycete ‘*Salinispora pacifica*’. Org Lett.

[b57] Piel J (2009). Metabolites from symbiotic bacteria. Nat Prod Rep.

[b58] Piel J (2011). Approaches to capturing and designing biologically active small molecules produced by uncultured microbes. Annu Rev Microbiol.

[b59] Pozzi R, Simone M, Mazzetti C, Maffioli S, Monciardini P, Cavaletti L (2011). The genus *Actinoallomurus* and some of its metabolites. J Antibiot.

[b60] Roemer T, Jiang B, Davison J, Ketela T, Veillette K, Breton A (2003). Large-scale essential gene identification in *Candida albicans* and applications to antifungal drug discovery. Mol Microbiol.

[b61] Simone M, Monciardini P, Gaspari E, Donadio S, Maffioli SI (2013). Isolation and characterization of NAI-802, a new lantibiotic produced by two different *Actinoplanes* strains. J Antibiot.

[b62] Singh SB, Phillips JW, Wang J (2007). Highly sensitive target-based whole-cell antibacterial discovery strategy by antisense RNA silencing. Curr Opin Drug Discov Devel.

[b63] Singh SB, Zink DL, Dorso K, Motyl M, Salazar O, Basilio A (2009). Isolation, structure, and antibacterial activities of lucensimycins D-G, discovered from *Streptomyces lucensis* MA7349 using an antisense strategy. J Nat Prod.

[b64] Tamura T, Ishida Y, Nozawa Y, Otoguro M, Suzuki K (2009). Transfer of *Actinomadura spadix* Nonomura and Ohara 1971 to *Actinoallomurus* spadix gen. nov., comb. nov., and description of *Actinoallomurus amamiensis* sp. nov., *Actinoallomurus caesius* sp. nov., *Actinoallomurus coprocola* sp. nov., *Actinoallomurus fulvus* sp. nov., *Actinoallomurus iriomotensis* sp. nov., *Actinoallomurus luridus* sp. nov., *Actinoallomurus purpureus* sp. nov. and *Actinoallomurus yoronensis* sp. nov. Int J Syst Evol Microbiol.

[b65] Thaker MN, Wang W, Spanogiannopoulos P, Waglechner N, King AM, Medina R, Wright GD (2013). Identifying producers of antibacterial compounds by screening for antibiotic resistance. Nat Biotechnol.

[b66] Tu Y, Yan B (2012). High-throughput fractionation of natural products for drug discovery. Methods Mol Biol.

[b67] Urban A, Eckermann S, Fast B, Metzger S, Gehling M, Ziegelbauer K (2007). Novel whole-cell antibiotic biosensors for compound discovery. Appl Environ Microbiol.

[b68] Wang H, Donk van der WA (2012). Biosynthesis of the class III lantipeptide catenulipeptin. ACS Chem Biol.

[b69] Wang J, Soisson SM, Young K, Shoop W, Kodali S, Galgoci A (2006). Platensimycin is a selective FabF inhibitor with potent antibiotic properties. Nature.

[b70] Wenzel SC, Müller R (2009). The impact of genomics on the exploitation of the myxobacterial secondary metabolome. Nat Prod Rep.

[b71] Zazopoulos E, Huang K, Staffa A, Liu W, Bachmann BO, Nonaka K (2003). A genomics-guided approach for discovering and expressing cryptic metabolic pathways. Nat Biotechnol.

[b72] Zengler K, Toledo G, Rappe M, Elkins J, Mathur EJ, Short JM, Keller M (2002). Cultivating the uncultured. Proc Natl Acad Sci USA.

[b73] Zhang C, Occi J, Masurekar P, Barrett JF, Zink DL, Smith S (2008). Isolation, structure, and antibacterial activity of philipimycin, a thiazolyl peptide discovered from *Actinoplanes philippinensis* MA7347. J Am Chem Soc.

